# Humans represent the precision and utility of information acquired across fixations

**DOI:** 10.1038/s41598-022-06357-7

**Published:** 2022-02-14

**Authors:** Emma E. M. Stewart, Casimir J. H. Ludwig, Alexander C. Schütz

**Affiliations:** 1 Department of Experimental Psychology, Justus-Liebig University Giessen, Otto-Behaghel-Str. 10F, 35394 Giessen, Germany; 2 School of Psychological Science, University of Bristol, Bristol, UK; 3 Allgemeine und Biologische Psychologie, Philipps-Universität Marburg, Marburg, Germany; 4 Center for Mind, Brain and Behaviour, Philipps-Universität Marburg, Marburg, Germany

**Keywords:** Human behaviour, Perception

## Abstract

Our environment contains an abundance of objects which humans interact with daily, gathering visual information using sequences of eye-movements to choose which object is best-suited for a particular task. This process is not trivial, and requires a complex strategy where task affordance defines the search strategy, and the estimated precision of the visual information gathered from each object may be used to track perceptual confidence for object selection. This study addresses the fundamental problem of how such visual information is metacognitively represented and used for subsequent behaviour, and reveals a complex interplay between task affordance, visual information gathering, and metacogntive decision making. People fixate higher-utility objects, and most importantly retain metaknowledge about *how much* information they have gathered about these objects, which is used to guide perceptual report choices. These findings suggest that such metacognitive knowledge is important in situations where decisions are based on information acquired in a temporal sequence.

## Introduction

The visual world is full of objects, each unique, and each rich in visual detail. In order to scrutinise these objects with higher-precision foveal vision, humans make sequences of saccades, sampling information with each fixation^1–4^. In such natural daily behaviour, humans make multiple saccades per second, and use the information that has been gathered across multiple fixations to inform decisions and actions. It is an open question as to what extent human observers have knowledge of the quantity and quality of perceptual evidence gathered across fixations and use this metaknowledge to guide their behaviour^5^. In daily life, representing the precision of the information one has about objects and locations in the world may be important when deciding on a course of action. For example, during an activity such as rock-climbing, one might need to find the most suitable object to use as the next hand-hold, based on visual information alone. Here one needs to not only represent the value, or utility of potential hand-hold-objects, but also the amount of confidence one has in the available visual information, to avoid choosing a bad object that is too small, slippery, or unstable, with potentially disastrous consequences. Such situations involve a number of processes: first, selecting potential high-utility objects to fixate, based on the particular affordance of the task; second, gathering higher-precision visual information about these objects using a sequence of saccades; third, maintaining a metacognitive representation about the precision of information gathered about each object; and fourth, choosing an object based on both its utility, and the metacognitive judgment about the precision of the information used to make this value-based comparison. It is unknown how such processes may interact, and in particular how humans represent and use metaknowledge about visual information acquired across sequences of saccades in such complex tasks.

**Figure 1:**
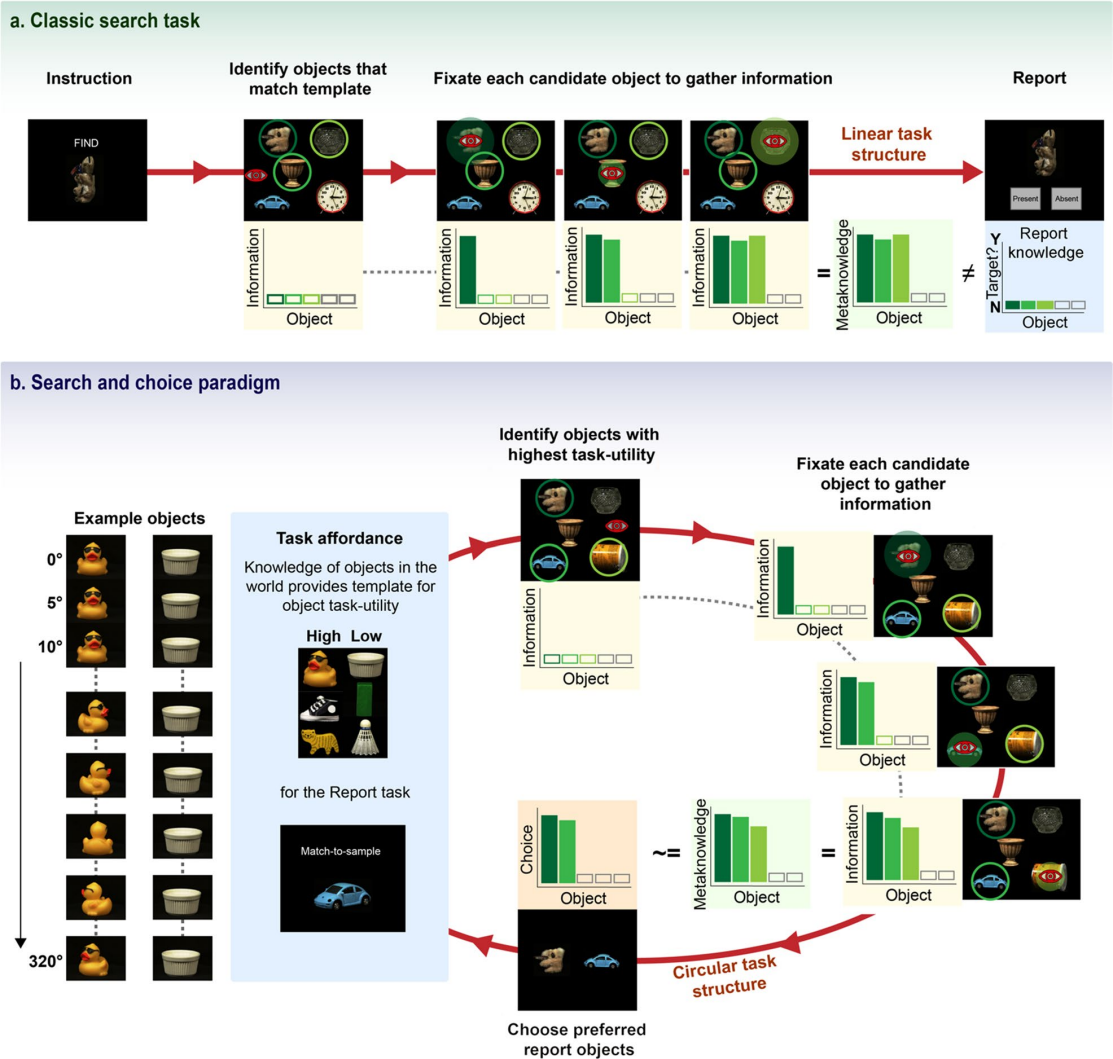
Comparison of classic search paradigms with the search and choice paradigm used in this study. (**a**) Example of a classic search paradigm, with a linear task structure: a search task is completed based on a template or instruction, but metaknowledge about the amount of information gathered for fixated objects is not necessary for, or probed in, the perceptual report task. (**b**) The framework for this experiment. In this framework, the constraints of the match-to-sample perceptual report task influence how information is sampled, and how this information is subsequently used. Here, the task structure is circular, because the requirements of the perceptual report task directly feed into the task affordance at the outset of the task: there are no specific search templates or instructions given at the start of each trial, rather the knowledge of the requirements of the eventual report task feed into the task affordances. The stimulus set comprises 577 unique objects, each with 360° viewpoints. Some objects, like the duck, are very easy to distinguish as they are rotated; others, like the ramekin, are very difficult to discriminate from one rotation to the next. Potential high-utility objects (in this case, those that are easier/more discriminable for the match-to-sample perceptual report task) are selected to be saccade targets; with each fixation, evidence about the orientation of the fixated object is gathered, along with a meta-representation of the degree of (un)certainty around this orientation: this can be roughly equated to perceptual confidence. Participants have to choose which objects they would prefer to report: they should choose those that they are more confident about (have more information about), and which, given the hypothesised search strategy, should also be easier to report. In this paradigm, the information gathered with each fixation is directly probed in the match-to-sample perceptual report task.

The question of how much metaknowledge humans have about their own oculomotor behaviour has been assessed in divergent studies investigating how much people know about their own eye movements. A number of authors have shown that people are unable to accurately report the locations of their fixations^5–7^ or recognize their own scanpaths from a visual search task^8^. However, other evidence suggests that people can remember their own fixations and better than chance^9^. More importantly, this memory for one’s own fixation behaviour seems to be particularly object-oriented^10–12^. People seem to retain a sparse memory representation of their own search history^13^, and the visual system itself seems to retain some implicit knowledge and memory of items and locations that have been fixated in visual search^14,15^. But while these studies assessed whether people knew *where* they looked, they did not assess whether people retained a representation of *how much information* they had gathered from each fixation. A highly influential model of optimal visual search assumes that the amount of information gathered at different locations of the search display is represented across multiple fixations^16^. However, how participants represent and use any metaknowledge of how much information they have gathered is unknown. In the studies gauging awareness of oculomotor behaviour, as well as standard search or information foraging paradigms, participants are asked to make reports such as whether a specific target is present or absent, where a particular target is located, or where they fixated^17^. However, these studies do not reveal whether participants retain metaknowledge about how much information they have gathered, and how they might use this metaknowledge to guide subsequent choices. While participants may gather information about various objects and locations during such search tasks, the subsequent report characteristics do not require participants to draw upon any metaknowledge they may have about how much information they have gathered (Fig. 1a). Many of the paradigms investigating what people know about their own eye movements also involve gauging participants’ *explicit awareness* of their own fixations: participants are asked to explicitly select objects, or locations they thought they had looked at, an approach which may under-estimate the amount of knowledge participants actually have about their fixation behaviour^18^. Therefore, in order to gauge how much information participants gather during fixations, and how much metaknowledge they have about this information, a different approach may be required than has been previously used.

Choice paradigms are commonly used in studies investigating value-based decision behaviour, where participants are asked to choose which of a number of stimuli they prefer. In many scenarios, these choices are based on both the subjective and relative value of these options, as well as viewing behaviour, with gaze amplifying the value of a choice option^19–23^. Oculomotor measures such as fixation duration and saccade vigour^24^ are predictive of choice (although these studies do not necessarily imply a causal role for gaze). Choice paradigms can also provide information about a participant’s level of uncertainty about a stimulus: when participants are able to choose which stimulus they prefer to report on, they consistently choose the less uncertain stimulus^25,26^. Report choice can also therefore be used as a proxy measure of perceptual confidence about a stimulus (Type 2 judgment), and can be used to probe knowledge about stimuli on a more metacognitive level. Confidence in perceptual decisions is dependent on factors such as the strength and duration of sensory inputs^27^, so in the context of this study, should relate to the higher-precision information gathered by fixations, and also fixation duration.

## Results

**Figure 2:**
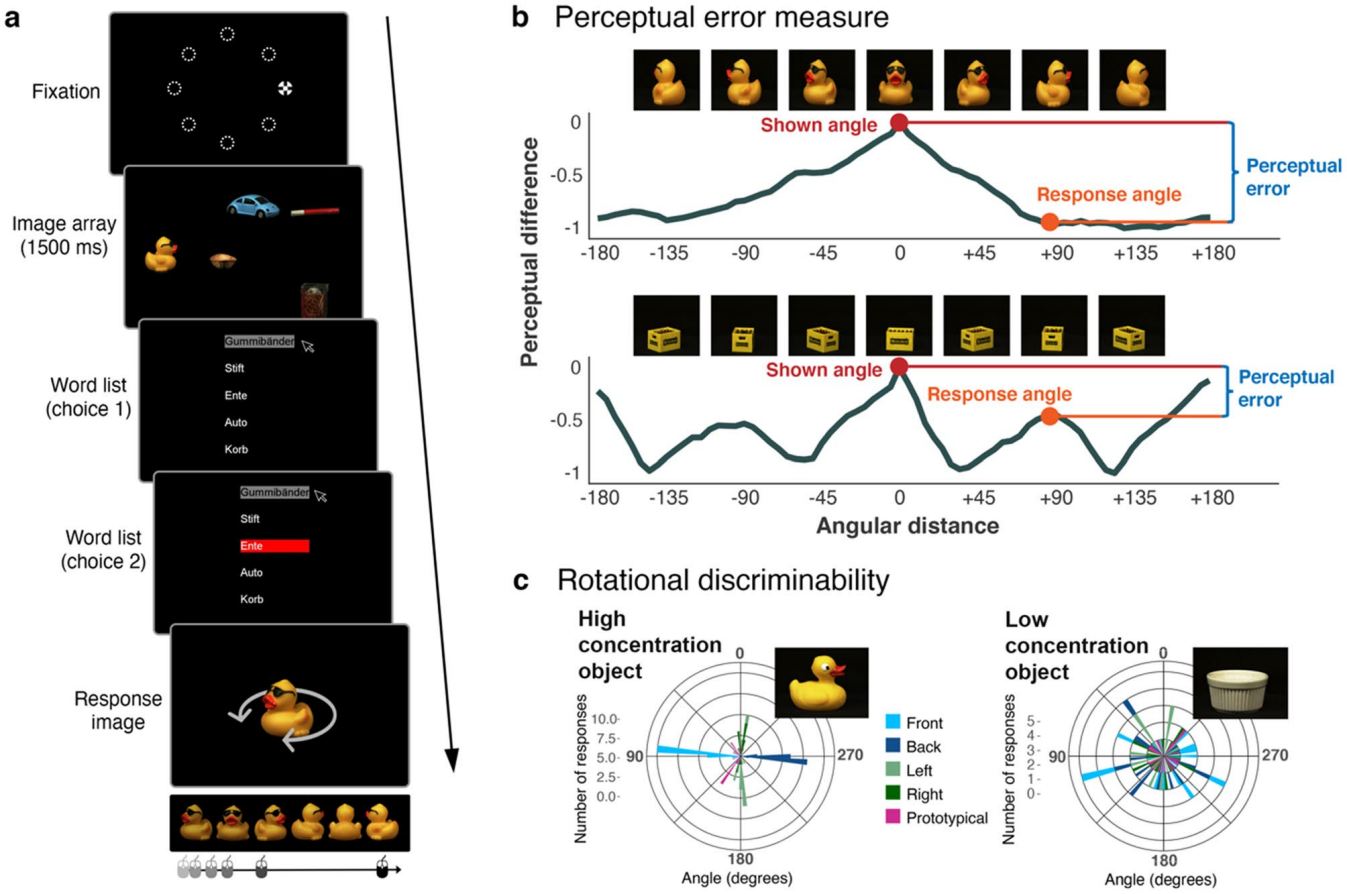
Methods. (**a**) Overview of experimental procedure: after fixation, an array of five objects at random rotations appeared for 1500 ms. Participants were free to inspect this array throughout this period. They were then asked to choose two objects they would prefer to report, and then rotate an on-screen image of one of the objects to match the shown rotation. The report object was more likely to be a chosen object, but non-chosen objects might also be probed. (**b**) Example of the perceptual error measure for two objects, based on low-level image properties: the top graph shows an object where angular difference corresponds almost linearly to perceptual dissimilarity. The bottom graph shows a regularly-shaped object where perceptual similarity peaks every 90° rotation. Perceptual error was calculated as the difference in perceptual similarity^28^ between shown and response angles: the same angular distance can result in a different perceptual distance for different objects. (**c**) Rotational discriminability (empirical) was measured in a separate, online study, and was estimated as the concentration of responses to axis labels for each object. Examples show a low and high concentration object, equating to low and high utility.

In this study we utilised a novel search, choice and report paradigm, in which participants free-viewed an array of five real-world objects for 1500 ms, and were then asked to choose which two objects they would prefer for a subsequent perceptual match-to-sample task (Figs. 1b, 2a). This task aimed to determine how the affordances of a specific task influence how humans use saccades to sample information, and how they use the metaknowledge about this sampled information to make report choices. We assume a theoretical framework, outlined in Fig. 1b, in which participants start a trial with the knowledge that they will have to complete a match-to-sample task at the end of that trial. Given the naturalistic and heterogenous nature of the presented objects, some items are inherently easier to report than others—these objects can be said to have greater task-utility. Participants should therefore use peripheral information to determine which objects in the trial have the greatest task-utility, and use saccades to sample more information from these objects. We hypothesise that participants retain a representation, or metaknowledge of the precision of the information gathered across fixations. If participants know how much information they have about each object, they should choose to report those objects that they have fixated and that they have more information about and can report more precisely. Therefore, report choice may be used as a measure of perceptual confidence. As a result, we predict links between fixation behaviour, information uptake, perceptual confidence (report choice) and perceptual error. Our primary hypotheses are therefore that (1) participants have more information for fixated items, compared to non-fixated items; (2) fixated items are more likely to be chosen for report; and (3) chosen and fixated items are reported more accurately. The properties of the objects used in this paradigm may also influence both report choice and fixation behaviour, such that participants should choose objects that are more suitable, or have greater *task utility* for the perceptual match-to-sample task, and should therefore fixate these easier objects in order to gather more information from them. Our secondary hypotheses therefore predict that objects with greater task-utility are more likely to be (4) chosen and (5) fixated. Note that when we refer to “more information”, or “more precise information”, we refer to the degree of (un)certainty about the object (or, its task-relevant dimension, namely orientation). We do not distinguish between the “quality” of information (i.e. higher acuity in foveal than peripheral vision), and “quantity” of information (i.e. more information can be obtained with longer fixation durations)—both direct fixation and prolonging fixation duration are ways of reducing uncertainty.

Results are split into two main sections: the first section relates to the question of how people use metaknowledge of the information they have acquired across fixations to make decisions about perceptual report. The second section relates to how people use intrinsic object properties and the task-utility of objects to guide these fixations and report choices.

### Metaknowledge and information

#### Fixations vs perceptual confidence vs performance

**Figure 3:**
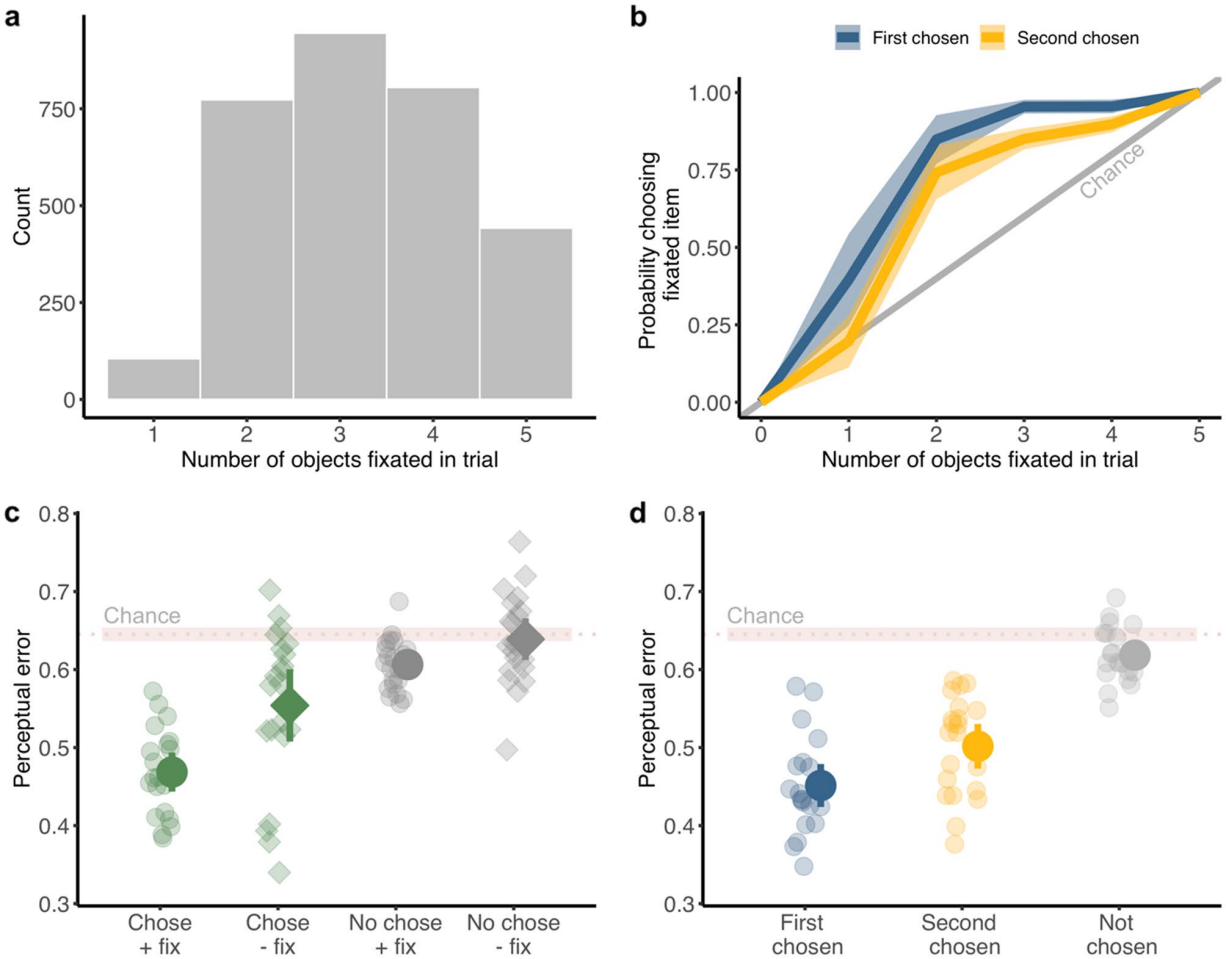
Fixations vs report choice vs performance. (**a**) Histogram of number of objects fixated in a trial, across all trials and participants. (**b**) Participants were more likely to choose objects that had been fixated, irrespective of how many items were fixated in a trial, for both first chosen (blue) and second chosen (yellow) objects. The grey diagonal line denotes chance performance for choosing a fixated item. (**c**) Perceptual error for report choice (chosen = green, not-chosen = grey) and fixation (fixated = circles, not-fixated = diamonds). (**d**) Perceptual error for first (blue), second (yellow) and not-chosen (grey) objects. (**c**, **d**) Light colors indicate individual participants and dark colors the mean across participants. The pink horizontal line denotes chance (for each trial, perceptual error was calculated using the perceptual similarity curve of one random object out of all 577 objects, and chance is the mean performance for this randomly-shuffled error). (**b**–**d**) All error bars are 95% confidence intervals.

First, we investigated how participants used the information they have acquired about the objects that were selected as saccade targets. Note that participants did not simply fixate all five objects, but were clearly selective. Figure 3a shows that on most trials 2–4 objects were fixated. The overall amount of time available to participants was limited to 1.5 s and this may have forced them to target more “promising” objects for fixation. To determine whether participants retained a representation of the precision of information acquired across the course of a trial, we compared fixations, report choice, and perceptual performance. If participants do retain a representation of this precision, they should choose objects that they have more information about as a result of fixation (hypothesis 2), which should also allow them to complete the behavioural task more accurately (hypothesis 3). More precise information should result from fixating an object, therefore performance should also be better for fixated objects. Figure 3b shows that this indeed the case: participants were more likely to choose objects they had fixated, irrespective of the number of objects fixated on a given trial, for both first and second chosen objects.

In addition, perceptual performance was better (lower perceptual error) for both chosen and fixated objects (Fig. 3c,d). A linear mixed model with fixed effect of fixation (fixated, not fixated) and report choice (chosen, not-chosen) and random by-participant intercepts was fitted to predict perceptual error on every trial. There was a significant main effect of fixation: F (1, 2995) = 23.65, p < 0.001; report choice: F (1, 2995) = 332.82, p < 0.0001; and an interaction between fixation and report choice: F (1, 2995) = 7.32, p = 0.0068.

#### How much information is needed to make choices?

**Figure 4:**
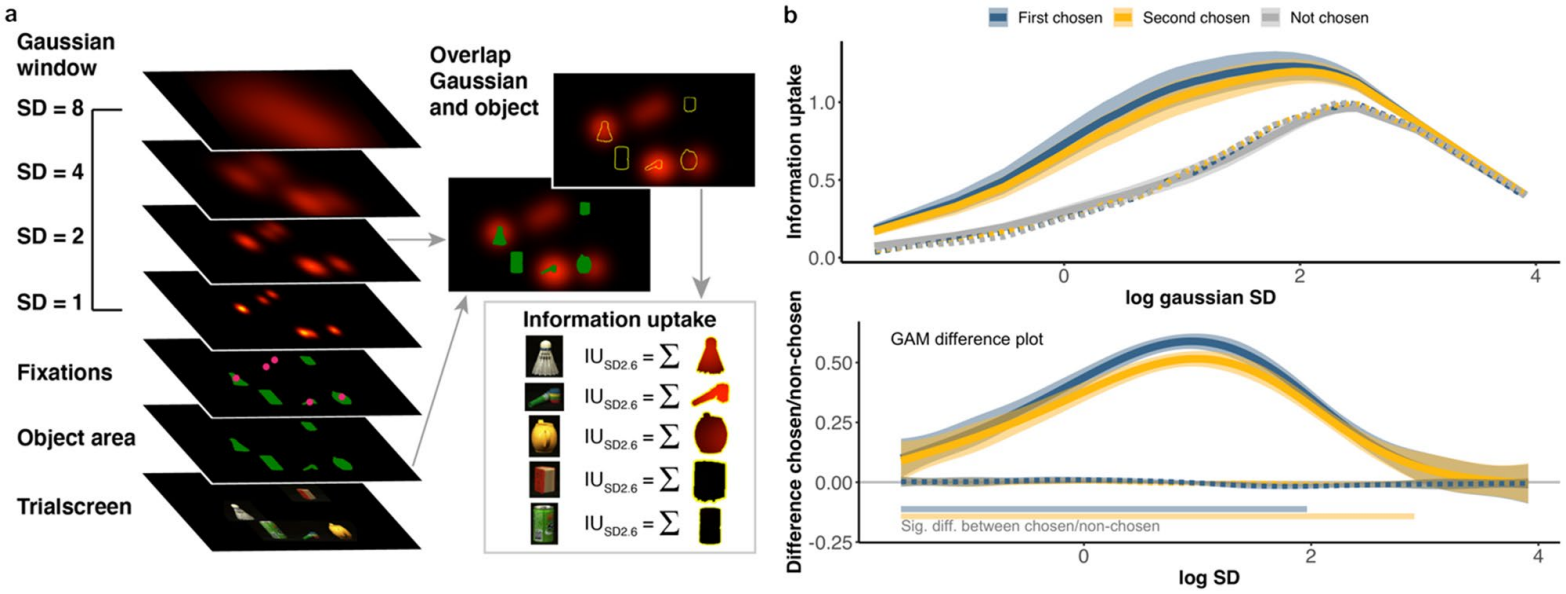
Overview of the information uptake model. (**a**) Information uptake was calculated by assuming Gaussian windows with different SDs around fixations for each trialscreen. Information uptake was calculated by summing the values of this Gaussian window across the object’s (x, y) pixel coordinates. Example overlap calculation is shown for the optimal window size, 2.6SD. (**b**) Top: Information uptake by report choice for first (blue), second (yellow) and not-chosen (grey) objects, by log SD of the Gaussian window. Dotted lines of the same colour indicate chance performance for each condition. Bottom: Difference between chosen and not-chosen objects from GAM models fitted to real (solid) and chance (dotted) data—note that the chance data overlap almost entirely. Error bars are 95% confidence intervals.

With each fixation, the visual system processes information from a certain area around the locus of fixation: this may occur in a very localised manner, where only high-acuity foveal information is processed; or may occur more broadly, where lower-acuity information is processed in a more diffuse manner from the entire visual field. We refer to the spatial extent of the information processed as an information uptake window. To investigate the link between fixation and report choice in more detail, we estimated the size of the “information uptake” window that was used to make report choices. We assumed that with every fixation, the visual system takes up information from a region of a certain size around the fixated point, and the precision of this information decreases with increasing distance from fixation. We additionally assumed that information uptake is a function of time, such that more information can be accumulated during a longer fixation^29,30^. We modelled this information uptake window as a Gaussian probability density function around each fixation, multiplied with the duration of that fixation:

1 $$Information\;uptake = \frac{1}{{\sqrt {2\pi \sigma^{2} } }}e^{{ - \frac{{\left( {x - \mu_{x} } \right)^{2} + \left( {y - \mu_{y} } \right)^{2} }}{{2\sigma^{2} }}}} \times fixation\;duration$$

where ${\mu }_{x}$ and ${\mu }_{y}$ are the *x* and *y* fixation coordinates and $\sigma$ is the circular standard deviation of the uptake window. As the size of this window is unknown, we varied $\sigma$ between 0.2° and 50° (from 0.2 to 3 in step sizes of 0.2, from 4 to 12 in step sizes of 1, and 20 and 50). At smaller values of $\sigma$, the assumption is that information is used from primarily foveal vision, including only information about objects close to fixation; at larger values of $\sigma$, the assumption is that information is parsed from a larger region of the visual field (Fig. 4a), including multiple objects also more distant from fixation. To determine how much information was taken-up from a particular object, we summed the Information Uptake values over the (*x*, *y*) coordinates occupied by that object across all fixations in a trial (Fig. 4a).

We then related this amount of information uptake for each object to report choice behaviour: if participants retained an accurate representation of how much information they had about each object, they should choose those they had more information about. Given the limited number of object locations used throughout the experiment, for larger window sizes we would already expect a significant amount of information uptake from the objects, even if fixation behaviour was entirely random. It is important to quantify this expected degree of information uptake for fixation behaviour that was not purposefully driven by the demands of the report choice task. We therefore computed a shuffled, baseline information uptake measure (see “Supp. materials”) (Fig. 4b, dotted lines). To quantify the difference between first, second, and non-chosen items across different size Gaussian windows, we used a generalised additive model (GAM) to describe information uptake by report choice and SD (similar to^31^). We compared the difference in information uptake between first and second chosen objects compared to non-chosen objects (non-chosen as baseline choice level). First, without taking window size into account, there was a significant main effect across all window sizes between non- and first-chosen objects (estimate = 0.39; std. error = 0.0085; *t* = 45.74; p < 0.0001); and between non- and second-chosen objects (estimate = 0.34; std. error = 0.0085; *t* = 40.32; p < 0.0001).

For the shuffled chance conditions (Fig. 4b, dotted lines), there was no significant difference between either non- and first chosen objects (estimate = − 0.0036; std. error = 0.0021; *t* = -1.72; p = 0.086); or between non- and second chosen objects (estimate = − 0.0035; std. error = 0.0021; *t* = − 1.66; p = 0.098). Therefore, the difference in information uptake between chosen and non-chosen is not an artefact arising from the window sizes adopted and the constrained object locations used throughout the experiment. We used difference smooths to compare the information uptake between chosen (first, second) and non-chosen objects across window size: the window sizes at which the 95% CI around these smooths do not contain 0 are considered significant (Fig. 4b, bottom). First-chosen objects had a different amount of information uptake to non-chosen objects for a SD range of 0.2–19.37 (non-log scale); second chosen objects differed from non-chosen for a SD range of 0.2–18.32. These results suggest that participants had more information about chosen objects compared to non-chosen objects, regardless of what assumptions we make about the size of the uptake window (up to very large windows of ~ 20° at least). Note that the peak difference between first-chosen and non-chosen, and between second-chosen and non-chosen were very similar (2.59° and 2.69°; in terms of full-width at half-height: 6.1° and 6.3°, respectively) (Fig. 4a “Overlap Gaussian and object” panel depicts a window with SD 2.6).

To determine whether the amount of information uptake for each object was predictive of perceptual error, beyond the categorical fixated/chosen predictors (as in Fig. 3c), we ran a linear mixed model as above, with information uptake (assuming a 2.6° window) as a factor. The mixed model contained a fixed effect of fixation (fixated, not fixated), report choice (chosen, not-chosen), and information uptake, with random intercepts by participant, and was fitted to predict perceptual error on every trial. The model was formulated such that the effect of information uptake was calculated given the effects of report choice and fixation (type I sequential sum of squares). There was a significant main effect of fixation: F (1, 2998) = 129.04, p = 0.0001; report choice: F (1, 2998) = 178.93, p < 0.0001; and more importantly information uptake: F (1, 2998) = 13.16, p < 0.0001, showing that information uptake accounted for performance beyond both report choice and fixation. There was also a significant interaction between information uptake, fixation, and report choice: F (1, 2998) = 6.83, p = 0.009. To investigate the effect of information uptake on perceptual error whilst controlling for fixation, we used this model to calculate the estimated difference in slopes of information uptake for fixated vs non-fixated objects (averaged across chosen/non-chosen). There was no difference in slopes between fixated and non-fixated objects: contrast Estimate [CI] − 0.05 [− 0.13 0.027], suggesting that the link between information uptake and perceptual error was independent of whether an object was fixated. When taking report choice into account, there is again no difference in slope between fixated and non-fixated chosen objects: estimate [CI] 0.053 [− 0.038 0.144], estimated slopes: non-chosen, non-fixated = 0.023 [− 0.047 0.11]; non-chosen, fixated = − 0.023 [− 0.07 0.025]. However, slopes differ for the chosen objects: estimate [CI] − 0.15 [− 0.28 − 0.028], estimated slopes: chosen, non-fixated = − 0.19 [− 0.31 − 0.06]; chosen, fixated = − 0.034 [− 0.05 − 0.011]. Specifically, when an object was chosen, there was a greater effect of information uptake on perceptual error for non-fixated objects. This indicates that the information uptake about an object may be a more accurate predictor of perceptual performance than fixation alone.

### Task constraints affect choice and fixation strategy

#### Report choice vs object features

**Figure 5:**
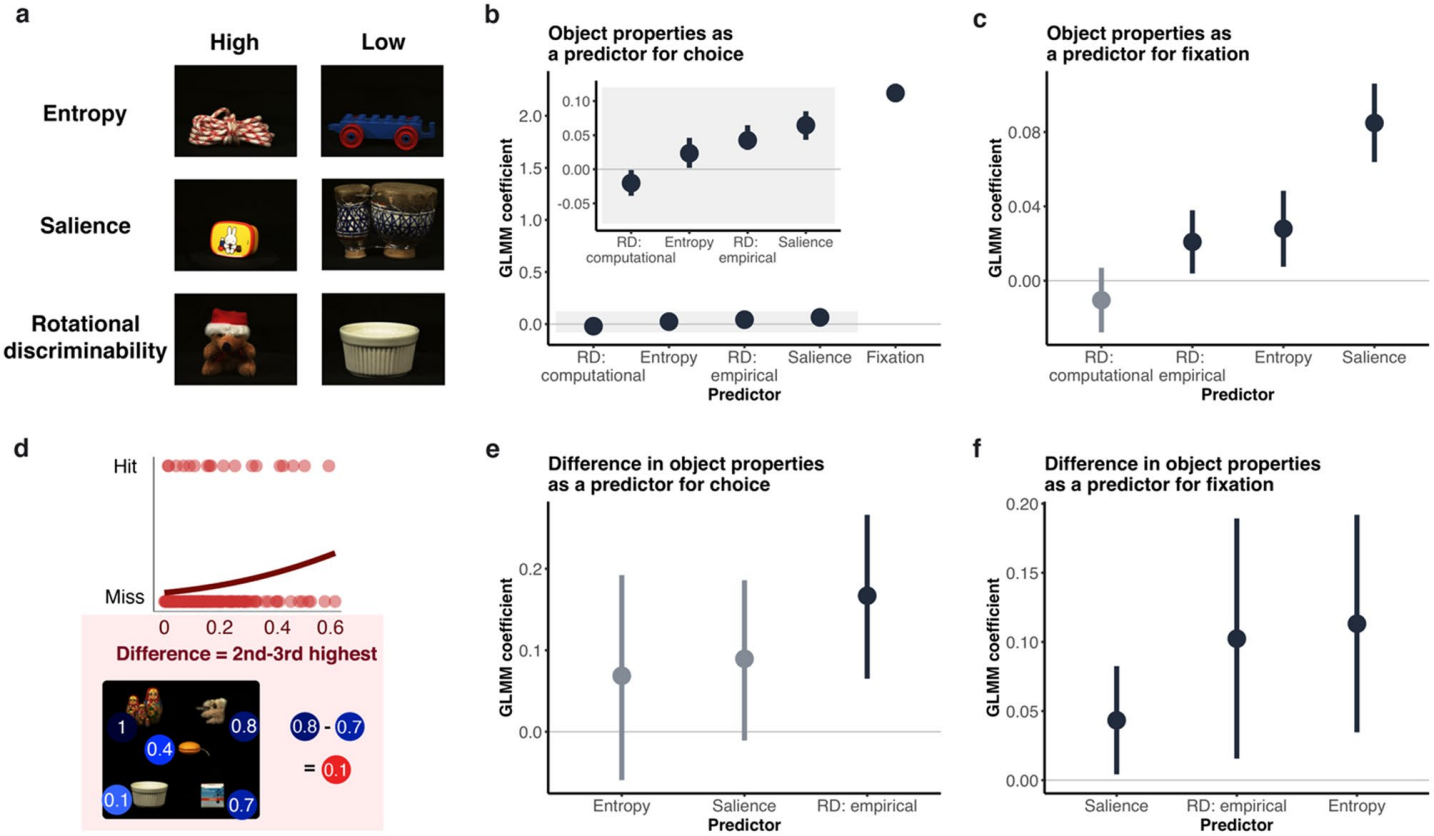
Object features as predictors of report choice and fixation. (**a**) Example images with high and low feature levels. (**b**) Model coefficient estimates from GLMM with object features as a predictor of report choice. The shaded area in the inset panel is a zoomed axis of the main plot. (**c**) Model coefficient estimates from GLMM with object features as a predictor of fixation. (**d**) Example of how the feature difference plots in E and F are calculated. The plot from an example observer shows the difference between the 2nd and 3rd highest rotational-discriminability of objects as a predictor for report choice, with the diagram depicting the difference calculation from a trial with arbitrary example values. Hits refer to trials where participants chose or fixated the two highest feature-value objects; miss refers to all other trials. (**e**) Model coefficient estimates from separate GLMMs for each feature, relating relative differences in object properties to report choices. (**f**) Model coefficient estimates from separate GLMMs for each feature, relating relative differences in object properties to fixation. (**b, c, e, f**) Error bars are 95% confidence intervals. Significant predictors are depicted in dark grey, insignificant in light grey.

To determine whether, on top of fixations, inherent object properties can also account for which objects were chosen in a trial (hypothesis 4), we related report choice decisions to a number of image properties: rotational discriminability (computational and empirical), salience, and entropy (Fig. 5a). Rotational discriminability was a measure of the utility of objects for the perceptual report task: if an object was more rotationally discriminable from one angle to the next, it would be easier to rotate it to match the shown angle (Fig. 2c), and therefore have more utility for the task. Participants should choose objects that were easier for the perceptual report task. Entropy was a measure of visual complexity, or variability in pixel values within each object, and salience was measured as an object feature that may affect report choice and fixation behaviour, but is not a measure of usefulness or information of an object.

For each object, in every trial, for every participant, we used a generalised linear mixed model to relate the binary outcome “chosen” (0 = not-chosen, 1 = chosen) to each object feature predictor (choice ~ fixation + rotational discriminability (computational) + rotational discriminability (empirical) + salience + entropy), with random by-subject intercepts. Fixation was the strongest predictor for report choice: estimate = 2.22, SE = 0.046, χ^2^ = 2322.99, p < 0.0001; followed by salience: estimate = 0.068, SE = 0.011, χ^2^ = 40.32, p < 0.0001; rotational discriminability (empirical): estimate = 0.026, SE = 0.009, χ^2^ = 8.01, p = 0.0046; and rotational discriminability (computational): estimate = − 0.02, SE = 0.01, χ^2^ = 4.8, p = 0.028 (note opposite direction as less peaks = higher rotational discriminability); and entropy: estimate = 0.022, SE = 0.011, χ^2^ = 3.93, p = 0.047 (Fig. 5b). This suggests that the best predictor of whether an object was chosen was whether it was fixated, but people were also more likely to choose objects that were salient, had higher rotational discriminability (therefore more utility for the task), or that had higher entropy.

To investigate whether the relationships between choice and object features were independent of fixation, we ran a second model which included an interaction between fixation and each feature predictor (choice ~ rotational discriminability (computational)*fixation + rotational discriminability (empirical)*fixation + salience*fixation + entropy*fixation). Model comparisons showed that adding interaction terms did not significantly improve the fit of the model (AIC_no-interction_ = 17,997; AIC_interaction_ = 18,002, χ^2^ = 2.86, p = 0.58). To further compare the effect of specific predictors on report choice whilst controlling for fixation, we calculated the estimated slope of each model covariate (feature) for fixated vs non-fixated objects. There was no difference in the slopes between fixated vs non fixated objects for salience: estimate [CI] 0.02 [− 0.03, 0.07]; RD (empirical): − 0.28 [− 0.07 0.016]; RD (theoretical): − 0.017 [− 0.06 0.03]; or entropy: − 0.03 [− 0.08 0.022]. This suggests that the effect of specific object features on report choice was independent of whether those objects were fixated or not.

If participants use a certain strategy to choose objects, for example choosing those that are more rotationally discriminable and therefore have a higher utility for the task, they should take into account the *relative* rotational discriminability of the objects in a particular trial. This relative judgement should be easier if there is a greater relative difference between higher and lower value objects. To see whether participants accounted for the relative magnitude of certain object properties, we looked at whether participants were more likely to choose the two objects with the highest magnitude of feature value, as the difference between the second and third highest-magnitude objects increased, or the relative difference between “higher” and “lower” magnitude objects increased (Fig. 5d). We tested the three object-property predictors: rotational discriminability/utility (empirical), salience, and entropy (the lack of variation in the rotational discriminability (computational) measure made it impossible to fit a model with this predictor, so we excluded it from this analysis), to see whether participants chose the most salient objects, or whether they chose objects based on their utility for the task, or the amount of information contained within an object. For each predictor, we ran a separate GLM with binary outcome of report choice (chosen, not-chosen) as predicted by the difference between second-highest and third-highest object for each trial and each subject (subject was included as a random effect). Wald chi-square tests showed a significant effect for rotational discriminability (empirical): estimate = 0.15, SE = 0.055, χ^2^ = 8.1, p = 0.0044; but no significant effect of salience: estimate = 0.07, SE = 0.064, χ^2^ = 3.19, p = 0.07; or entropy: estimate = 0.068, SE = 0.064, χ^2^ = 1.14, p = 0.28 (Fig. 5e). This demonstrates that participants chose objects based on their relative utility, rather than salience or entropy. This suggests a higher-level strategy whereby participants can assess how suitable each object is for the perceptual discrimination task, and then choose objects based on this relative judgement.

#### Fixation vs object features

Participants were more likely to choose objects that are better for the perceptual report task; this may suggest that they also used a strategy to fixate higher-utility objects in order to extract more information from them for the eventual perceptual report task (hypothesis 5). We therefore also investigated whether inherent object properties influenced fixation strategies. As above, for each object in every trial for every participant, we used a generalised linear mixed model to relate the binary outcome “fixated” (0 = not fixated, 1 = fixated) to each object feature predictor (fixation ~ rotational discriminability (computational) + rotational discriminability (empirical) + salience + entropy), with random effect of subject. Salience was the strongest predictor for fixation: Estimate = 0.085, SE = 0.01, χ^2^ = 63.07, p < 0.0001; followed by entropy: Estimate = 0.03, SE = 0.01, χ^2^ = 7.03, p = 0.008; rotational discriminability (empirical): Estimate = 0.02, SE = 0.009, χ^2^ = 5.056, p = 0.025; and rotational discriminability (computational): Estimate = -0.01, SE = 0.009, χ^2^ = 1.3, p = 0.25 (Fig. 5c). This suggests that, after salience, the largest predictors of fixation were entropy and rotational discriminability (empirical), with higher entropy objects (those that contained more detailed information), and more rotationally discriminable (higher utility) objects being fixated more often. We further investigated whether the relative magnitude of objects’ features affected which objects were fixated: as with the report choice analysis, we looked at whether participants were more likely to fixate the two objects with the highest feature magnitude as a function of the difference in feature strength between the second and third highest objects. There was a significant effect of relative rotational discriminability: Estimate = 0.1, SE = 0.04, χ^2^ = 5.6, p = 0.018; relative entropy: Estimate = 0.11, SE = 0.04, χ^2^ = 7.9, p = 0.0048; and relative saliency: Estimate = 0.043, SE = 0.02, χ^2^ = 4.71, p = 0.03 (Fig. 5f). As with report choice, this suggests that participants accounted for the relative strength of features in objects, and used this to guide saccades, and that the relatively utility of objects, or the amount of information contained within an object, may play more of a role than their relative salience.

## Discussion

This study provides insights into how information is acquired, represented, and used in a complex visually-guided choice task. The results showed that humans retain an accurate representation of the precision of information acquired across a sequence of saccades. Participants had higher perceptual confidence and lower perceptual error for objects that they had fixated, and thus had higher-precision information for. Participants also chose objects based on relevant features, such as their relative utility for the perceptual report task (as judged independently by a different sample of participants in a different experiment).

This novel paradigm provides strong evidence that humans know how much information they have gathered about objects across sequences of fixations. In the context of studies investigating whether humans can explicitly remember their own oculomotor behaviour^7–10,12^, this metaknowledge may be functionally more useful than retaining knowledge of exact fixation locations: knowing whether the available information about an object is reliable is important when this information informs subsequent decisions and actions. Our model of information uptake also suggests that humans consider primarily foveal information to make choices, suggesting that choices are based on the higher-precision, foveal information that is gained from fixations. However, when the influence of fixations is accounted for, intrinsic object features also determined which objects people chose, regardless of whether they were fixated or not. This suggests that participants did not solely rely on information from fixated objects, but to a lesser extent also processed and used peripherally-viewed object information, potentially accumulating evidence for both fixated and peripherally-viewed objects in parallel over the course of multiple fixations^32,33^.

The task affordances in this paradigm showed a secondary behavioural outcome: participants could account for the relative inherent utility of objects when making choices. Here, utility was not measured as an explicit rating or reward, but as an implicit measure, where items with a higher utility were those that made the perceptual task easier (operationalized as “rotational discriminability”). Participants were more likely to choose, and fixate objects with higher utility relative to all objects presented, demonstrating that they retained an accurate representation of not only the internal precision of information they had about each object, but also the relative utility of each object in the display. Similar links between fixations, choice, and subjective value have been found in studies where participants had to choose food items that they had given overt explicit value ratings for^20,21,30^, where participants made decisions and saccades based on both the strength of purely sensory evidence and explicit value of potential targets^34–37^. The measure of utility in this study differs from these previous studies, however, as no explicit valuation was given for objects, and participants had to infer the utility of each object for the perceptual report task based on visual information alone: in this sense these findings bridge studies on perceptual and value-based decision-making^20,30,34,36,38^, by considering the *inherent* perceptual utility of objects, where the utility of each option is based on how its visual properties make an object more or less suitable for a purely perceptual task, independent of the behaviour of the participant. It is of course possible that participants can select the best objects without this choice being accompanied by a metacognitive, subjective experience of confidence. However, that seems unlikely given the wealth of evidence that people have knowledge about their own level of uncertainty in perceptual decision making^26^. Nevertheless, it is a possibility that any subjective experience of confidence that accompanies selection of the items for report, plays no causal role in the selection of the objects and is constructed post-hoc, after the selection has been made (e.g. post-decision models of confidence such as^39^). However, we refrain from making inferences about whether people have explicit access to their own internal, subjective states—this is why this paradigm uses an implicit measure of confidence based on choice.

This paradigm also allowed us to relate how fixation strategy might be driven by task affordances. Participants tended to fixate more salient items (Fig. 5a)^40–42^. Note that many studies have reported that salience is not the greatest predictor for fixations^43–46^, but these findings generally apply to natural scene viewing. In our study, where discrete objects are placed in an array on a black screen, the role of saliency might be higher due to a lack of context or contextual meaning. However, once salience was accounted for, people also seemed to fixate the higher-utility (i.e. more rotationally-discriminable) objects that would be more appropriate for the perceptual report task, and those that had higher entropy, or contained a higher variability of visual information within the shown view of the object. Indeed, these measures had a larger effect than saliency when considering the *relative* magnitude of features in an object in the context of the whole visual scene—i.e. participants were more likely to fixate the objects with relatively higher utility and entropy, but not those with relatively higher salience. This suggests that when accounting for features that relate directly to the task affordance (such as utility), it is the relative magnitude of these features that is important.

The link between fixations and object features is interesting in the context of influential saccade sampling theories such as those of Najemnik and Geisler^16,47^, which suggest that humans fixate objects and regions with higher uncertainty, in order to gain information. In this experiment one might expect people to fixate a “difficult” object in order to gain as much information as possible, in case they have to report this object. However, the strategy of looking at “better” items in this case suggests a more confirmatory approach to choosing fixation targets, where participants’ sampling strategies are biased toward preferred, or more valuable items (similar to the MAP strategy rejected by Najemnik and Geisler^47^, but see also Eckstein et al.^48^). Why might this be the case? The first explanation may again relate to the constraints of the paradigm itself: when sampling visual information for a subsequent report choice, the strategy of fixating objects based on the expected utility of the object for the task could outweigh any low-level perceptual consequences such as information gain (where one might expect participants to fixate more difficult objects to gather more information—this strategy might arguably be used if the task constraints differed, i.e. if participants were not able to choose a preferred report item, or if the contingency between choice and probe were changed, so that non-chosen items had to be reported more often). The sampling strategy seen in this study may also reflect studies showing that a later reward associated with a decision may influence saccade strategies leading up to that decision^48^. This strategy is reminiscent of confirmation bias in economic decision making, where participants’ sampling strategies are biased toward preferred, or more valuable items^38,49,50^. However, in the context of this study, object utility was not based on monetary reward, but on perceptual information. The second explanation proposes a strategy where participants account for both utility and visual information gain^16,47^. The utility of an object may represent an upper bound on the amount of information that can be gained from fixating that object: more information can be gained (or more uncertainty reduced) from high- than low-utility items. For example, for objects such as the ramekin (Fig. 2c), fixation would not provide any more beneficial information about the object for the task. Maximum information gain is achieved through fixating an object, but when the upper bound on potential information gain is low (as with low-utility items), the information that can be gained from fixation may not necessarily increase compared to the information gained from peripheral viewing.

One limitation of our study is that we did not analyse to what extent fixations and report choices were determined by semantic information about the objects. Semantic information has been shown to be an important determinant of fixations in real-world scenes^45,51–56^. However, we do not believe that semantic information played a major role in our study for three reasons: first, our displays were composed of unrelated, isolated objects without any (semantic) scene context. Second, we used a purely perceptual task that could be achieved even without recognizing a particular object. Third, semantic information only guides attention when searching for a semantically related object^57^. Since participants were not searching for a particular object in our task, guidance by semantic information should not be activated.

A related issue, relevant to both semantics and utility, is whether participants used some inherent metaknowledge of the objects themselves as a predictor of rotational discriminability. We cannot, for example, know whether participants knew from experience and encounters with objects in the real world, that a rubber duck may have higher utility for the task than a uniform glass bowl, or whether they used low-level visual cues to judge utility. Additionally, while we see convincing evidence for the effect of utility, it is certainly possible that our measure of utility could also correlate with a different visual cue—with such heterogenous stimuli there could be boundless possibilities for what this cue might be.

Gathering visual information to make a visually-guided choice about the best object for a task is a non-trivial process: this study reveals a complex interplay between task affordance, visual information gathering, and metacognitive decision making, and suggests first, that humans can retain and use metacognitive knowledge about the precision of visual information gathered across sequences of saccades; and second that task affordances result in participants using the inherent perceptual utility of objects to guide both fixations and choices.

## Methods

For more detailed methods please refer to “Supplementary material”.

### Participants

Twenty-one participants aged 18–30 (16 female, 5 male) took part in the experiment for money or course credits. This sample size is similar to previous studies on reporting oculomotor behaviour^7,12^. Ethics approval was obtained from the local ethics commission of the Department of Psychology of Marburg University (proposal numbers 2015-35k and 2020-43k), participants provided informed consent, and experiments were conducted in accordance with the Declaration of Helsinki (1964).

### Stimuli

Stimuli came from the Amsterdam Library of Object images (ALOI)^58^, which comprises real-world, everyday objects, photographed from 72 viewpoints (360°, in 5° rotational increments) on a black background (Fig. 1b). We selected a subset of objects that were easily recognisable and nameable for our subjects, which resulted in 577 objects being used in the experiment. The images appeared at random locations on an equally spaced triangular grid (such that x and y distances in the grid were equal), with a maximum horizontal eccentricity of 15°, and vertical eccentricity of 10° from the centre of the screen. The fixation cross was a white combination of a bulls-eye and a cross-hair shape^59^, and never appeared at the same location as an image in a trial.

### Procedure

To start the experiment, participants fixated on a cross which appeared randomly at one of eight locations equally spaced on an imaginary circle with radius 8° from screen centre, and pressed the space bar (Fig. 2a). After a random delay between 500 and 1000 ms, five images appeared on the screen for subjects to view freely. After 1500 ms, the images disappeared and a list of the names of the five objects appeared. Subjects had to use the mouse to select two objects that they preferred to report on. After selection, a response image appeared. The response image could be an image that was both chosen and fixated, chosen but not fixated, not-chosen and fixated, or neither chosen nor fixated (see “Supplementary material” for fixation classification details). Participants were told that it was more likely that the response image would be one they chose. Participants used a mouse to rotate the response image to match the rotation they saw, and clicked to confirm. We aimed for chosen objects to be presented at double the probability of non-chosen items. Due to differing choice/fixation behaviour between participants, it was not always possible to achieve consistent probabilities for report items: the mean and SD of response items across participants for each choice/fixation combination are: chosen, fixated: 0.56 (0.04); chosen, not-fixated: 0.07 (0.04); not-chosen, fixated: 0.19 (0.08); not-chosen, not-fixated: 0.18 (0.09). Each participant completed 150 trials; an object could only be repeated after 70 trials to avoid memory interference. Note that participants were given no instructions as to which objects they should look at, or which objects might be easier for the task. They were given no specific instructions in regards to their viewing behaviour. Participants were unaware of how perceptual error was calculated, and were given no explicit feedback on their performance during the experiment.

### Analysis

#### Performance—perceptual similarity

To determine perceptual performance, we used a perceptual similarity metric rather than calculating error as angular error between the shown and reported object orientations: some objects, for example a cube-shaped box, look very similar with every 90° rotation, so measuring perceptual similarity between rotations gives a more accurate representation of perceptual error than measuring pure angular error (compare, for example, Fig. 2b top to bottom where the same angular error can result in different perceptual error for different objects). To do this, we used the Perceptual Similarity measure of Neumann and Gegenfurtner^28^, which uses low-level image features such as colour and spatial frequency to calculate the perceptual similarity of two images. We calculated perceptual error between the shown rotation of the response image and all other rotations of that image, and normalised these values between 0 and − 1 per object, such that the comparison of an image to itself had a similarity score of 0, and the most dissimilar rotation had a score of − 1 (normalisation did not affect overall results). The perceptual error was the absolute similarity score on this perceptual similarity curve at the reported object rotation (Fig. 2b).

#### Object properties analyses

We used the following methods to quantify the properties of the objects used in the experiment. For full analysis details see “Supplementary material”.

##### Rotational discriminability

A key measurement was rotational discriminability, which was used to quantify how easy each object was for the match-to-sample task. This measurement was used as a proxy for object utility. We quantified rotational discriminability with both an empirical, and computational measure. Rotational discriminability (computational) was based on the perceptual similarity metric described above (Fig. 2b), and was defined as the number of peaks on that curve for an object’s shown rotation. An object such as the duck has a single peak, and is thus more discriminable from each angle compared to an object such as the yellow box. Rotational discriminability (empirical) was measured via a separate, online study. Participants were instructed to rotate objects to match the cardinal axis labels: front, back, left, right, prototypical. To quantify rotational discriminability, we examined the distribution of responses for each cardinal axis label separately. If an object was highly rotationally-discriminable, there should be high agreement between participant responses to an axis label; if an object was not rotationally-discriminable, there should be lower agreement between participant responses to an axis label. To quantify this, we measured the mean resultant length of responses for each axis label (between 0 for highly dispersed and 1 for highly concentrated; Fig. 2c): an object with high concentration has distinct angles, and therefore has high rotational discriminability; an object with low concentration looks similar from one angle to the next, and therefore has low rotational discriminability.

##### Salience

Low-level saliency for each object was computed using the CovSal saliency metric^60^. As we were interested in the relative saliency of objects as they appeared in a trial, salience was calculated on a trial-wise basis, such that each object in a given trial was given a salience score in comparison to the other objects in the trial.

##### Entropy

Shannon’s entropy^61^ was calculated to quantify the visual complexity of an object, or the variability in pixel values contained within the image. An object with higher entropy, therefore more pixel variability may provide may fine-grain visual cues to perform the visual matching task than an object with little variability.

### Statistical analyses

All statistical analyses were conducted in R. For mixed-model analyses, random effects structures are described in the results section.

#### Linear mixed model (LMM) details

Linear mixed models were fit using the nlme package^62^. Pairwise comparisons and estimated marginal means were calculated using the emmeans package^63^. For linear mixed models, fixed effects of fixation (fixated, not fixated) and choice (chosen/not-chosen or first, second, not-chosen) were categorically coded using treatment coding, with the baseline level coded as not-chosen or not fixated. Individual trials which were influential to the regression fit for a particular analysis were removed (Cook’s distance^64^). Where models had heterogenous variance (differing variability across different levels of a factor), model comparisons were used to determine the best-fitting variance structure (i.e. different variance allowed for different factor levels). Significance was determined using a Type I (sequential sum of squares) analysis of variance. F-statistics were calculated using the estimated error variance from the full form of the model. All model estimates are included in the SI.

#### Generalised additive models (GAM) details

GAMs were used for Information Uptake modelling analyses. GAMs were fit with the package mgcv^65^. All GAMs had a thin plate regression spline smoother applied to log Gaussian SD, for each choice condition (first, second, non-chosen), and random smooth for subjects.

#### Generalised linear mixed model (GLMM) details

GLMMs were fitted using the lme4 package^66^. GLMMs were used to relate object features to choices and fixations. Feature predictors were scaled by the mean level of the predictor across all trials, divided by 2 SD^67^ to normalise the large differences in scale between predictors and improve model fit. To ensure collinearity between predictors did not affect model estimations, we calculated the variance inflation factor (VIF) for each predictor. For fixation, VIF = 1.00; rotational discriminability (empirical), VIF = 1.03; rotational discriminability (theoretical), VIF = 1.09; entropy VIF = 1.46; salience VIF = 1.4. Given the low VIF values for all predictors, it is unlikely that predictor collinearity affected estimates^68^. Significance was determined using a Wald chi-square test.

## Supplementary Information

Supplementary Information.

## Data Availability

Data is available at 10.5281/zenodo.5266520.

## References

[1] Schutz AC, Braun DI, Gegenfurtner KR (2011). Eye movements and perception: A selective review. J. Vis..

[2] Hayhoe MM (2016). Vision and action. Annu. Rev. Vis. Sci..

[3] Eckstein MP (2011). Visual search: A retrospective. J. Vis..

[4] Gegenfurtner KR (2016). The interaction between vision and eye movements†. Perception.

[5] Marti S, Bayet L, Dehaene S (2015). Subjective report of eye fixations during serial search. Conscious Cogn..

[6] Võ ML-H, Aizenman AM, Wolfe JM (2016). You think you know where you looked? You better look again. J. Exp. Psychol. Hum. Percept. Perform..

[7] Kok EM, Aizenman AM, Võ ML-H, Wolfe JM (2017). Even if I showed you where you looked, remembering where you just looked is hard. J. Vis..

[8] Clarke ADF, Mahon A, Irvine A, Hunt AR (2016). People are unable to recognize or report on their own eye movements. Q. J. Exp. Psychol..

[9] Foulsham T, Kingstone A (2013). Where have eye been? Observers can recognise their own fixations. Perception.

[10] Irwin DE, Zelinsky GJ (2002). Eye movements and scene perception: Memory for things observed. Percept. Psychophys..

[11] Zelinsky GJ, Loschky LC (2005). Eye movements serialize memory for objects in scenes. Percept. Psychophys..

[12] Clarke ADF, Coco MI, Keller F (2013). The impact of attentional, linguistic, and visual features during object naming. Front. Psychol..

[13] Dickinson CA, Zelinsky GJ (2007). Memory for the search path: Evidence for a high-capacity representation of search history. Vis. Res..

[14] McCarley JS, Wang RF, Kramer AF, Irwin DE, Peterson MS (2003). How much memory does oculomotor search have?. Psychol. Sci..

[15] Peterson MS, Kramer AF, Wang RF, Irwin DE, McCarley JS (2001). Visual search has memory. Psychol. Sci..

[16] Najemnik J, Geisler WS (2005). Optimal eye movement strategies in visual search. Nature.

[17] Wolfe JM (2021). Guided Search 6.0: An updated model of visual search. Psychon. B Rev..

[18] Wu C-C, Wolfe JM (2018). A new multiple object awareness paradigm shows that imperfect knowledge of object location is still knowledge. Curr. Biol..

[19] Gluth S, Kern N, Kortmann M, Vitali CL (2020). Value-based attention but not divisive normalization influences decisions with multiple alternatives. Nat. Hum. Behav..

[20] Smith SM, Krajbich I (2018). Gaze amplifies value in decision making. Psychol. Sci..

[21] Thomas AW, Molter F, Krajbich I, Heekeren HR, Mohr PNC (2019). Gaze bias differences capture individual choice behaviour. Nat. Hum. Behav..

[22] Callaway F, Rangel A, Griffiths TL (2021). Fixation patterns in simple choice reflect optimal information sampling. Plos Comput. Biol..

[23] Krajbich I, Rangel A (2011). Multialternative drift-diffusion model predicts the relationship between visual fixations and choice in value-based decisions. Proc. Natl. Acad. Sci..

[24] Reppert TR, Lempert KM, Glimcher PW, Shadmehr R (2015). Modulation of Saccade Vigor during value-based decision making. J. Neurosci..

[25] Barthelmé S, Mamassian P (2009). Evaluation of objective uncertainty in the visual system. Plos Comput. Biol..

[26] Mamassian P (2015). Visual confidence. Annu. Rev. Vis. Sci..

[27] Yeung N, Summerfield C (2012). Metacognition in human decision-making: Confidence and error monitoring. Philos. Trans. R. Soc. B Biol. Sci..

[28] Neumann D, Gegenfurtner KR (2006). Image retrieval and perceptual similarity. ACM Trans. Appl. Percept. (TAP).

[29] Cassey TC, Evens DR, Bogacz R, Marshall JAR, Ludwig CJH (2013). Adaptive sampling of information in perceptual decision-making. PLoS ONE.

[30] Krajbich I, Armel C, Rangel A (2010). Visual fixations and the computation and comparison of value in simple choice. Nat. Neurosci..

[31] Stewart EEM, Schütz AC (2019). Transsaccadic integration is dominated by early, independent noise. J. Vis..

[32] Busemeyer JR, Gluth S, Rieskamp J, Turner BM (2019). Cognitive and neural bases of multi-attribute, multi-alternative, value-based decisions. Trends Cogn. Sci..

[33] Ludwig CJH, Davies JR, Eckstein MP (2014). Foveal analysis and peripheral selection during active visual sampling. Proc. Natl. Acad. Sci. USA.

[34] Summerfield C, Koechlin E (2010). Economic value biases uncertain perceptual choices in the parietal and prefrontal cortices. Front. Hum. Neurosci..

[35] Schütz AC, Trommershäuser J, Gegenfurtner KR (2012). Dynamic integration of information about salience and value for saccadic eye movements. Proc. Natl. Acad. Sci..

[36] Gold JI, Shadlen MN (2001). Neural computations that underlie decisions about sensory stimuli. Trends Cogn. Sci..

[37] Summerfield C, Tsetsos K (2015). Do humans make good decisions?. Trends Cogn. Sci..

[38] Summerfield C, Tsetsos K (2012). Building bridges between perceptual and economic decision-making: Neural and computational mechanisms. Front. Neurosci-switz..

[39] Pleskac TJ, Busemeyer JR (2010). Two-stage dynamic signal detection: A theory of choice, decision time, and confidence. Psychol. Rev..

[40] Kienzle, W., Wichmann, F., Schölkopf, B. & Franz, M. A nonparametric approach to bottom-up visual saliency. in *Advances in Neural Information Processing Systems 19: Proceedings of the 2006 Conference* 689–696 (2007).

[41] Koehler K, Guo F, Zhang S, Eckstein MP (2014). What do saliency models predict?. J. Vis..

[42] Parkhurst D, Law K, Niebur E (2002). Modeling the role of salience in the allocation of overt visual attention. Vis. Res..

[43] Einhauser W, Spain M, Perona P (2008). Objects predict fixations better than early saliency. J. Vis..

[44] Tatler BW, Hayhoe MM, Land MF, Ballard DH (2011). Eye guidance in natural vision: Reinterpreting salience. J. Vis..

[45] Henderson JM, Hayes TR (2017). Meaning-based guidance of attention in scenes as revealed by meaning maps. Nat. Hum. Behav..

[46] Underwood G, Foulsham T, Humphrey K (2009). Saliency and scan patterns in the inspection of real-world scenes: Eye movements during encoding and recognition. Vis. Cogn..

[47] Najemnik J, Geisler WS (2008). Eye movement statistics in humans are consistent with an optimal search strategy. J. Vis..

[48] Eckstein MP, Schoonveld W, Zhang S, Mack SC, Akbas E (2015). Optimal and human eye movements to clustered low value cues to increase decision rewards during search. Vis. Res..

[49] Shimojo S, Simion C, Shimojo E, Scheier C (2003). Gaze bias both reflects and influences preference. Nat. Neurosci..

[50] Jonas E, Schulz-Hardt S, Frey D, Thelen N (2001). Confirmation bias in sequential information search after preliminary decisions: An expansion of dissonance theoretical research on selective exposure to information. J. Pers. Soc. Psychol..

[51] Võ ML-H, Wolfe JM (2013). The interplay of episodic and semantic memory in guiding repeated search in scenes. Cognition.

[52] Henderson JM, Hayes TR (2018). Meaning guides attention in real-world scene images: Evidence from eye movements and meaning maps. J. Vis..

[53] Wolfe JM, Horowitz TS (2017). Five factors that guide attention in visual search. Nat. Hum. Behav..

[54] Torralba A, Oliva A, Castelhano MS, Henderson JM (2006). Contextual guidance of eye movements and attention in real-world scenes: The role of global features in object search. Psychol. Rev..

[55] Nuthmann A, Henderson JM (2010). Object-based attentional selection in scene viewing. J. Vis..

[56] Võ ML-H, Boettcher SE, Draschkow D (2019). Reading scenes: How scene grammar guides attention and aids perception in real-world environments. Curr. Opin. Psychol..

[57] Nuthmann A, de Groot F, Huettig F, Olivers CNL (2019). Extrafoveal attentional capture by object semantics. PLoS ONE.

[58] Geusebroek J-M, Burghouts GJ, Smeulders AWM (2005). The Amsterdam library of object images. Int. J. Comput. Vision.

[59] Thaler L, Schutz AC, Goodale MA, Gegenfurtner KR (2013). What is the best fixation target? The effect of target shape on stability of fixational eye movements. Vision. Res..

[60] Erdem E, Erdem A (2013). Visual saliency estimation by nonlinearly integrating features using region covariances. J. Vis..

[61] Shannon CE (1948). A mathematical theory of communication. Bell. Syst. Tech. J..

[62] Pinheiro, J., Bates, D., DebRoy, S., & Sarkar, D., & R Core Team. *nlme: Linear and nonlinear mixed effects models*. (2020). Retrieved from https://CRAN.R-project.org/package=nlme.

[63] Lenth, R., Buerkner, P., Herve, M., Love, J., Riebl, H., & Singmann, H. *emmeans: Estimated marginal means, aka least-squares means*. (2020). Retrieved from https://CRAN.R-project.org/package=emmeans.

[64] Cook RD (1977). Detection of influential observation in linear regression. Technometrics.

[65] Wood, S. N. Generalized additive models. (2017). 10.1201/9781315370279.

[66] Bates D, Mächler M, Bolker B, Walker S (2015). Fitting linear mixed-effects models using lme4. J. Stat. Softw.

[67] Gelman A, Hill J (2006). Data Analysis Using Regression and Multilevel/Hierarchical Models (Analytical Methods for Social Research).

[68] Fox J, Weisberg S (2019). An R Companion to Applied Regression.

