# Supplementary material for: Humans represent the precision and utility of information acquired across fixations
Source: Sci Rep. 2022 Feb 14;12:2411. doi: 10.1038/s41598-022-06357-7 (PMC8844410; doi:10.1038/s41598-022-06357-7)
Supplement: Supplementary file 1 — Supplementary Information. [file 41598_2022_6357_MOESM1_ESM.pdf]

Supplementary Information for

Humans represent the precision and utility of information acquired across fixations

Emma E.M. Stewart[1*], Casimir J.H. Ludwig[2], Alexander C. Schütz[3,4]
1 Department of Experimental Psychology, Justus Liebig University Giessen, Giessen, Germany
2 School of Psychological Science, Bristol University, Bristol, UK
3 Allgemeine und Biologische Psychologie, Philipps-Universität Marburg, Marburg, Germany
4 Center for Mind, Brain and Behavior, Philipps-Universität Marburg, Marburg, Germany

*Emma E.M. Stewart
Abteilung Allgemeine Psychologie
FB06 Psychologie und Sportwissenschaften
Justus-Liebig-Universität Gießen
Ph: +4915161548990
Email: Emma.Stewart@psychol.uni-giessen.de

Supplementary methods

Participants

All participants were naïve as to the purposes of the experiment and had normal or corrected-to-normal vision.

Equipment

Stimuli were presented on a VIEWPixx Monitor (VPIXX Technologies Inc., Saint-Bruno, Canada) with a resolution of 1920 x 1080 pixels, at a viewing distance of 60cm. To maintain the typical appearance of the objects, the standard non-linear gamma function of the monitor was used without a gamma correction. The luminance of black, gray and white pixels was 0.38, 17.16 and 105.50 cd/m^2, respectively. Eye movements were recorded monocularly using a desktop-mounted Eyelink 1000 (SR Research Ltd., Ottawa, Canada) with a sampling rate of 1000Hz. Participants were calibrated using a standard nine-point calibration and validation procedure. Stimuli were presented using Matlab 2016a, and the Psychtoolbox (1, 2). Responses were recorded using a standard keyboard and mouse.

Stimuli

The size of the base rectangle of each image on a black background was 8.2 x 6.1 degrees, however the actual area covered by image vs background varied between objects. On each trial, objects were randomly chosen so that each object was from a unique superordinate (i.e. fruits/vegetables) and subordinate (i.e. strawberry) category. Figure S1 shows the counts of objects in each superordinate category. A full list of objects will be made available at https://doi.org/10.5281/zenodo.5266520.

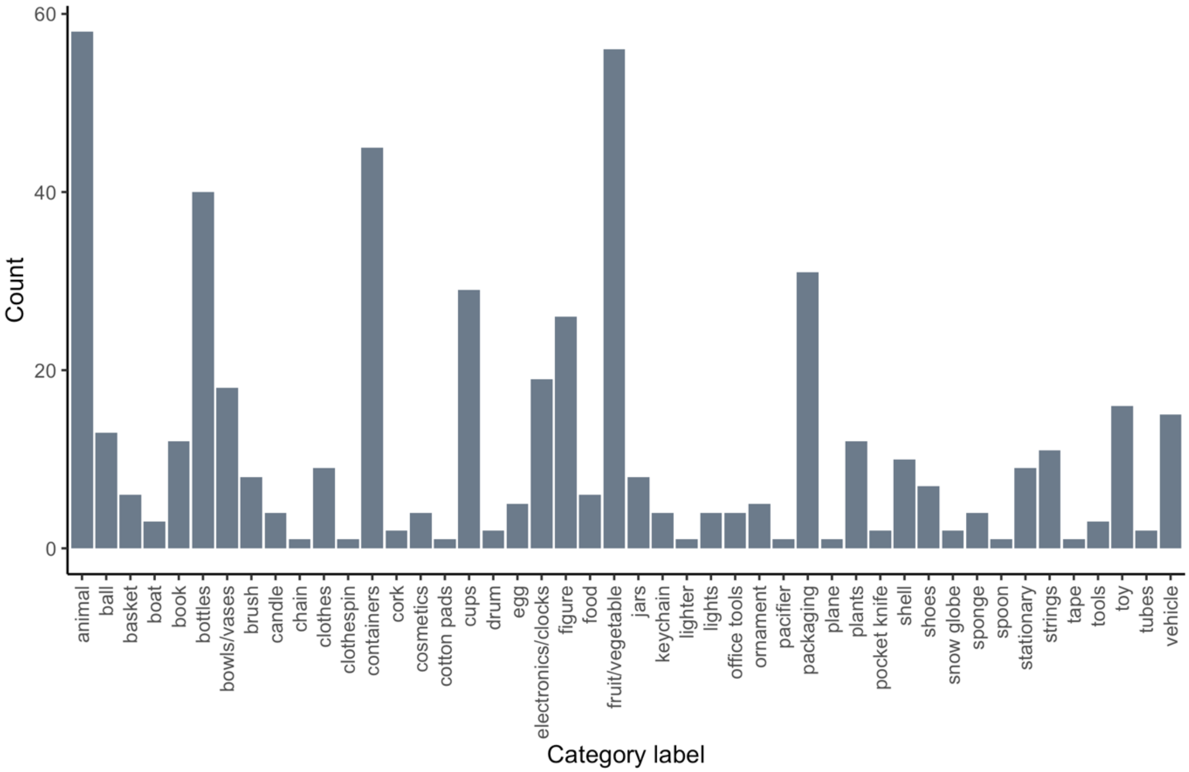

Figure S1. Category labels for the objects use in the experiment.

Procedure

Object selection: once the first selection was made that word was highlighted in red. The position of the mouse cursor was indicated with a grey box highlighting the word nearest the cursor.

Supplementary analyses

Object properties analyses

Object properties – rotational discriminability (empirical)

We conducted an online study to obtain an empirical measure of rotational discriminability. For this online study, 235 participants aged 18-69 (7 unspecified age; 114 female, 116 male, 2 other, 3 unspecified gender) were recruited through the online recruitment platform Prolific and were given monetary reimbursement. Participants were shown fifty randomly selected objects. For each object, they were instructed to use the computer keyboard to make five perceptual reports by rotating the object to match the cardinal axis labels: front, back, left, right, prototypical. Each participant completed all five rotations for fifty randomly-selected objects. We excluded trials where the data demonstrated low-effort responses: where the participant did not rotate the object on the response screen, or where reaction time was less than 500 ms or more than 100 seconds. This resulted in 11,837/12,208 (97%) trials being included for analysis. The mean number of ratings per object was 22.6, SD = 4.2. We used concentration around the prototypical axis as the measure of empirical rotational discriminability as this axis had the most even spread of concentration values. Results were consistent irrespective of the axis used to define this measure.

Object properties – rotational discriminability (computational)

We used the perceptual similarity metric of Neumann & Gegenfurtner (3) to determine a computational measure of the "rotational discriminability" of each object – in other words how different the object was from one viewpoint to the next. We used the Matlab function "findpeaks" to characterise the perceptual similarity curve for each object in a trial (perceptual similarity was calculated for each trial, based on the actual angle that was presented in that trial): this function defines the width and height of peaks in a signal, and the number of peaks. We constrained the function with a parameter defining minimum peak height as main peak height/4. We defined computational rotational discriminability as the number of peaks defined by this function, with higher values (more peaks) corresponding to a less discriminable object.

Object properties – salience

Low-level salience was calculated using the CovSal saliency metric, using covariates and means with no centre-bias (4). Salience scores were calculated as the sum activation for an object's base region.

Object properties – entropy

Entropy was calculated for each object and rotation in every trial to determine the amount of variability in visual information conveyed by each object. Shannon's entropy (5) is defined as:

$$H(X) = -\sum_{i=1}^{n} P(x_i) log_2 P(x_i) \quad \text{(Eqn. S1)}$$

Where P is the probability of each possible present pixel value (x_i) occurring in the image. The higher the entropy, the higher the visual complexity, or variability in pixel values contained within the image.

Fixation classification

Within the experiment, an object was classified as fixated if gaze fell within the base rectangle area for that image, however, the heterogenous nature of the images in the ALOI means that although the size of the base rectangle of the image on the background was identical for each image, the area of that rectangle that was covered by the image versus background differed between objects. Thus, to more accurately classify fixated objects in further analyses, for each image and object rotation, we determined the area that was actually occupied by the object. We distinguished "object" pixels from "background" pixels by defining a routine in Matlab which first defined all black pixels (RGB = [0 0 0]) as NaN, and non-black pixels as 1, and then refined the object from background noise and shadows by using the inbuilt function bwconncomp to locate any clusters of non-black pixels and incrementally convert the smallest cluster to NaN, until no clusters of <100 pixels were left. After this procedure, to account for objects with interior black pixels, for each row, the minimum and maximum non-NaN pixels were located and all pixels between those points were define as object pixels. For 54/577

objects this routine did not work (translucent or glass objects were difficult to quantify), and object area was manually defined for these objects using Photoshop. We calculated the centre of mass of the resulting object area and determined a “fixation zone” around this centre. This fixation zone was calculated as a circular area half the maximum object dimension (horizontal or vertical) + 1.6°. An object was classified as fixated if a fixation occurred within its fixation zone. Fixations were determined by the Eyelink 1000+ algorithm (velocity threshold = 22°/s, acceleration threshold = 3800°/s^2).

Exclusions

No trials were excluded, as participants were not given explicit instructions on viewing behaviour.

Supplementary results

Perceptual error and choice order

To investigate whether choice order affected perceptual error (Figure 3D), we used a linear mixed model predicting perceptual error, with a fixed effect of choice (not-chosen vs first vs second chosen objects) with random by-participant intercepts, and variance structure accounting for differing variance across each level of choice. There was a significant effect of choice: $F(2,3119) = 133.17$, $p <0.0001$, with post-hoc multiple comparisons revealing a difference between not-chosen and first-chosen: $t(3119) = 15.63$, $p = <0.0001$; first-chosen and second-chosen: $t(3119) = -4.43$, $p <0.0001$; and second-chosen and not-chosen: $t(3119) = -10.48$, $p<0.0001$.

Fixation order vs choice

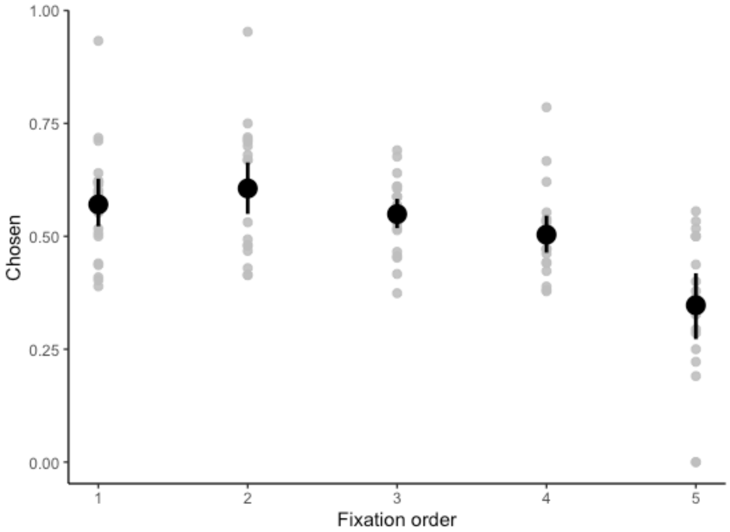

Figure S2 *- mean proportion of chosen items (0 = not chosen, 1=chosen) by fixation order (1 = 1st fixated etc), across participants.*

To determine whether the probability of choosing an object might be driven by any primacy or recency effects, we investigated whether participants were more likely to choose an item depending on the order it was fixated. A repeated measures ANOVA for choice~fixation_order suggested a significant effect of order: $F(4,97) = 12.85$, $p<0.0001$, however post-hoc pairwise comparisons with a Holm correction for multiple comparisons show that this effect is driven entirely by the fact that the "5" level, in other words the 5th and last object to be viewed, was chosen less:

Table S1 – post-hoc multiple comparison results.

Contrast, fixation order number	*estimate*	*SE*	*df*	*t.ratio*	*p.value*
1 - 2	-0.035	0.038	97	-0.92	0.73
1 - 3	0.021	0.038	97	0.55	0.73
1 - 4	0.067	0.039	97	1.72	0.44
1 - 5	**0.223**	**0.039**	**97**	**5.66**	**<0.0001**
2 - 3	0.057	0.038	97	1.48	0.57
2 - 4	0.102	0.039	97	2.63	0.06
2 - 5	**0.259**	**0.039**	**97**	**6.55**	**<0.0001**
3 - 4	0.046	0.039	97	1.17	0.73
3 - 5	**0.202**	**0.039**	**97**	**5.12**	**<0.0001**
4 - 5	**0.156**	**0.040**	**97**	**3.91**	**0.0012**

Given that the proportion of trials in which 5 items were fixated was quite low (see Figure S2 above), we are hesitant to make any inferences from this data – and indeed this experiment was not designed to optimally investigate primacy/recency effects.

Information uptake

We computed this baseline level of information uptake as follows. For a every trial for each participant, we took the observed sequence of fixation location and duration, and computed the information uptake for objects from a trialscreen from a different, randomly chosen trial from the same participant. The information uptake for chosen and non-chosen objects was then averaged per participant, as with the observed, non-shuffled data.

Choice and relative object features

When comparing relative object features, we only used the measure of rotational discriminability (empirical). Because the rotational discriminability (computational) measure is based on the number of peaks in the perceptual similarity curve for an object, in many trials the number of peaks for the five objects would be relatively uniform (for example [1 1 1 2 2]). In these trials, it is impossible to calculate the difference between the 2nd and 3rd best objects, and there is only a small variation in differences for the remaining trials.

Object feature correlations

Table S2. Feature value correlations.

Variable	RD (empirical)	Salience	RD (computational)
Salience	.14**		
RD (computational)	-.13**	-.16**	
Entropy	.09**	.54**	-.28**

Note. * indicates $p < .05$. ** indicates $p < .01$.

Note that the measures of entropy and rotational discriminability are weakly numerically correlated and so also may be related in the amount of information each measure provides to the participant for the task. An object with a large amount of variation in pixel values, or detail (for example, a can of tomatoes with distinct writing or symbols on the label) may have high entropy, and will also have a high utility for the rotation task, as participants would be able to match these fine-grained details.

Full model estimates for LMMs

Table S3. Fixation vs choice vs performance (Figure 3C).
Model specification: error ~ choice*fix, random = ~1|subject, weights = (~1|choice*fix)

	Perceptual error ~ fixation, choice			
Predictors	*Estimates*	*std. Error*	*CI*	*df*
(Intercept)	0.65	0.01	0.63 – 0.68	2995.00
choice [1]	-0.10	0.02	-0.14 – -0.07	2995.00
fix [1]	-0.03	0.01	-0.05 – -0.00	2995.00
choice [1] * fix [1]	-0.06	0.02	-0.10 – -0.02	2995.00
Observations	3019			

Table S4 - Model specification: error ~ fixated*chosen*information_uptake, random = ~1|subject, weights = (~1|chose*fix)

	Perceptual error ~ fixation, choice, information uptake			
Predictors	*Estimates*	*std. Error*	*CI*	*df*
(Intercept)	0.64	0.01	0.61 – 0.66	2998.00
fix [1]	0.00	0.03	-0.05 – 0.05	2998.00
chose [1]	-0.03	0.02	-0.08 – 0.02	2998.00
information_uptake	0.03	0.04	-0.05 – 0.11	2998.00
fix [1] * chose [1]	-0.10	0.04	-0.17 – -0.03	2998.00
fix [1] * information_uptake	-0.05	0.05	-0.14 – 0.04	2998.00
chose [1] * information_uptake	-0.22	0.07	-0.36 – -0.07	2998.00
(fix [1] * chose [1]) * information_uptake	0.21	0.08	0.05 – 0.36	2998.00
Observations	3026			

Supplementary references

1. D. H. Brainard, Spatial vision. *The psychophysics toolbox* 10, 433–436 (1997).

2. D. G. Pelli, The VideoToolbox software for visual psychophysics: transforming numbers into movies. *Spatial Vision* 10, 437–442 (1997).

3. D. Neumann, K. R. Gegenfurtner, Image retrieval and perceptual similarity. *ACM Transactions on Applied Perception (TAP)* 3, 31–47 (2006).

4. E. Erdem, A. Erdem, Visual saliency estimation by nonlinearly integrating features using region covariances. *J Vision* 13, 11–11 (2013).

5. C. E. Shannon, A mathematical theory of communication. *Bell Syst Technical J* 27, 379–423 (1948).
